# The Neurology of Menopause

**DOI:** 10.1007/s11910-026-01506-1

**Published:** 2026-07-24

**Authors:** Hannah J. Roeder, Enrique C. Leira

**Affiliations:** 1 Department of Neurology, University of Iowa, 200 Hawkins Dr, Iowa City, IA 52242 USA; 2 Departments of Neurology, Neurosurgery, and Epidemiology, University of Iowa, Iowa City, USA

**Keywords:** Menopause, Estrogen, Stroke, Epilepsy, Migraines, Multiple Sclerosis

## Abstract

**Purpose of review:**

Menopause is a neuroendocrine process with important implications for neurological health. This review examines the complex, multidirectional relationships between menopause and major central nervous system conditions, including stroke, migraine, epilepsy, multiple sclerosis, Parkinson’s disease, and Alzheimer’s disease.

**Recent findings:**

Menopause may mark an inflection point for vascular injury, neurodegeneration, and disability accumulation in several neurological conditions. Perimenopausal hormonal fluctuations may exacerbate migraines and seizures in susceptible women. Research increasingly supports the critical window hypothesis for estrogen therapy and highlights the importance of distinguishing reproductive and chronological aging when interpreting neurologic trajectories.

**Summary:**

Menopause represents an important transition for many conditions. Hormone therapy has heterogenous effects with cognitive benefit in premature ovarian insufficiency, harm related to stroke risk in older women, and greater uncertainty in other conditions. Therefore, individualized risk-benefit assessment is essential. Research gaps remain significant, and menopause should be a priority area for neurologic research.

## Introduction

Menopause, a normal developmental milestone experienced by approximately half the population, is the permanent cessation of menstruation resulting from loss of follicular activity. Menopause is diagnosed 12 months after the final menstrual period, in the absence of other physiologic or pathologic cause [1]. Induced menopause may occur secondary to surgical procedures or medical interventions that cause cessation of ovarian function. The Stages of Reproductive Aging Workshop + 10 (STRAW + 10) criteria broadly categorizes reproductive, menopausal transition, and postmenopausal periods [2]. Menstrual cycles of variable length and increasing intervals of amenorrhea characterize the menopausal transition. Perimenopause, when vasomotor symptoms are most likely to occur, encompasses both the menopausal transition and the early postmenopausal period. In the United States, the average age of menopause is 52 years old. With average life expectancy at 81.4 years, over one-third of the average US woman’s life is expected to occur after menopause [3].

**Fig. 1:**
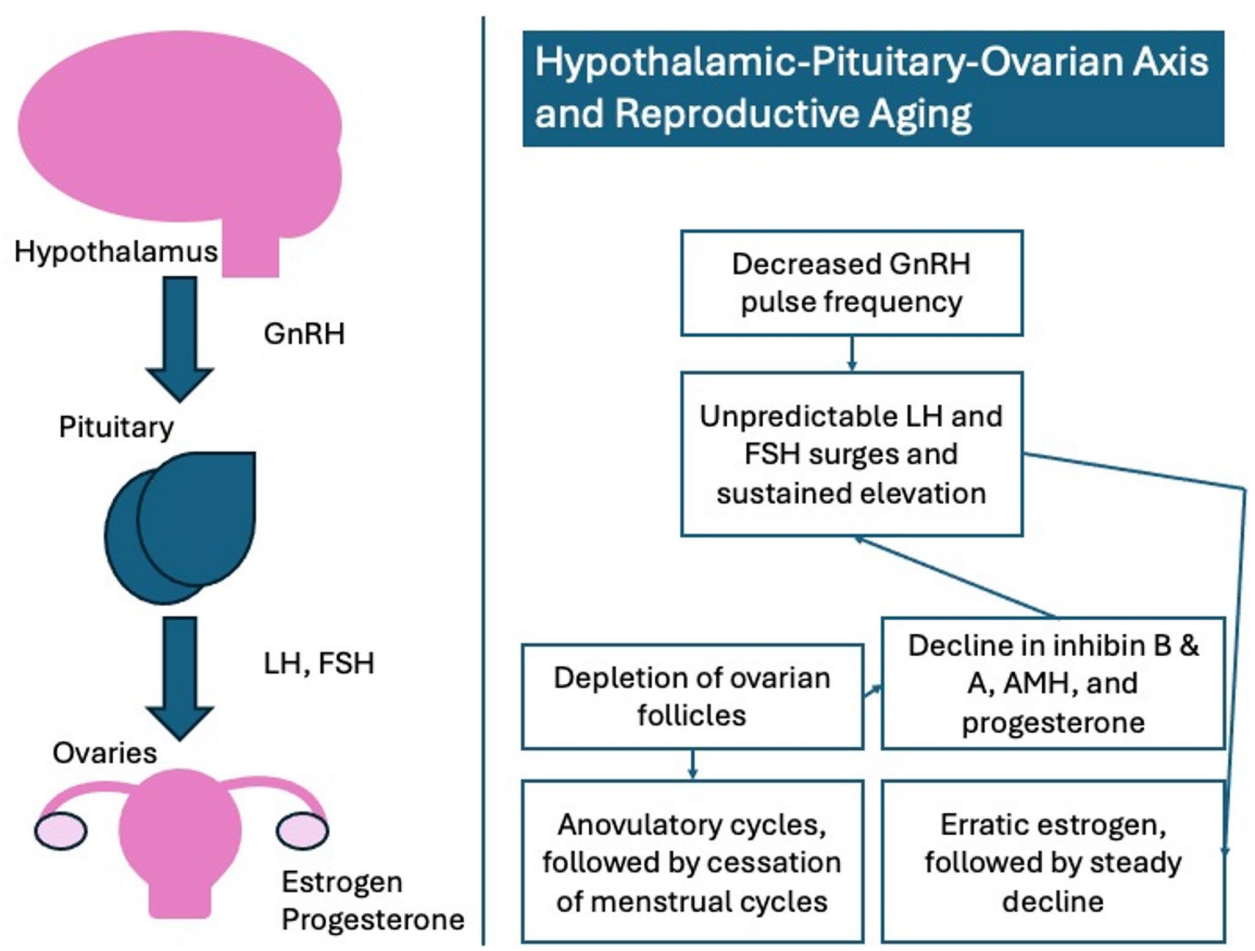
Figure 1: Hypothalamic-Pituitary-Ovarian Axis and Reproductive AgingDuring the menopausal transition, the HPO axis undergoes significant changes. Ovarian follicular depletion and age-related neuroendocrine changes at the hypothalamic and pituitary levels leads to progressive breakdown of the coordinated HPO feedback loops that regulate reproductive function. These changes have significant implications for many neurological diseases. GnRH (gonadotropin-releasing hormone), LH (luteinizing hormone), FSH (follicle-stimulating hormone), AMH (anti-mullerian hormone)

Critical changes in the hypothalamic-pituitary-ovarian axis occur during the perimenopausal period (Fig. 1). Many common perimenopausal symptoms (including hot flashes, night sweats, insomnia, fatigue, depression, and anxiety) reflect that reproductive aging is a neuroendocrine process. The health of the brain and ovaries are inextricably linked. This review explores the complex interactions between menopause and several central nervous system (CNS) conditions. The relationships are multidirectional. Menopause and the treatment of menopausal symptoms influence the clinical course and management of neurologic disease. Neurologic disease and its treatment impact reproductive health. The narrative review specifically focuses on the interactions between menopause and stroke, migraine, epilepsy, multiple sclerosis, Parkinson’s disease, and Alzheimer’s disease.

## Stroke

Sex differences in stroke risk change throughout the life course. Young adult women have a higher stroke hazard compared to young adult men, largely driven by pregnancy-related and hormonal factors [4]. Women have a lower incidence of stroke compared to men for most of middle adulthood; however, menopause is an inflection point for a sharp increase in stroke risk in women, which roughly doubles in the decade following menopause and continues to rise throughout the postmenopausal period (Table 1) [5]. Eventually, stroke risk in older women equalizes or surpasses the risk in men [6–8].

**Table 1:** Changes in Neurological Diseases during the Menopausal Transition and Post-Menopause

	Menopausal Transition	Post-menopause
Stroke	• Stroke risk increases	• Risk continues to climb
Migraine	• May exacerbate migraine without aura, particularly with history of menstrual migraines	• Migraine without aura tends to improve • Migraine with aura and isolated aura more likely to continue
Epilepsy	• May exacerbate seizures in WWE with history of catamenial seizures	• Improves for many WWE with prior catamenial seizures • Highly variable for other seizure types
MS	• May represent inflection point towards fewer relapses yet greater disability accumulation	• Ongoing immunosenescence combined with inflammaging
PD	• Hormonal and immune changes contribute to PD risk	• When most diagnoses occur, more common in men than women
AD	• Hormonal and metabolic changes increase vulnerability to developing AD pathology	• When most diagnoses occur, more common in women than men

*MS* (multiple sclerosis),* PD* (Parkinson’s Disease),* AD* (Alzheimer’s Disease)

Several, interacting pathophysiologic changes account for accelerated vascular risk during the menopausal transition, many of which are independent of chronological aging and driven by the loss of endogenous estrogen’s protective effects on the vasculature, lipid metabolism, blood pressure regulation, glucose homeostasis, and the coagulation cascade [9]. Estrogen decline leads to decreased nitric oxide synthesis, endothelial dysfunction, and disruption of flow-mediated dilation [10]. Estrogen withdrawal activates the renin-angiotensin-aldosterone system promoting maladaptive vascular remodeling and hypertension [11]. Deleterious lipid changes also accelerate atherogenesis [12]. Other metabolic changes, such as insulin resistance and weight gain, may be driven more by chronological aging but coincide with menopause and contribute to stroke risk [9]. Anti-inflammatory and neuroprotective effects of endogenous estrogens also decline. Other hormonal changes (e.g., androgens, progesterone) may contribute to postmenopausal stroke risk but the mechanisms are less understood.

As the loss of endogenous estrogens contributes to stroke risk, investigators in the 1990s hypothesized that exogenous estrogen replacement may prevent ischemic stroke; however, a series of major trials suggested otherwise. The Women’s Estrogen for Stroke Trial (WEST) was a randomized controlled trial of oral estrogen therapy for secondary stroke prevention in postmenopausal women that did not impact stroke recurrence [13]. The Heart and Estrogen/progestin Replacement Study (HERS) was a randomized controlled trial of estrogen plus progestin in postmenopausal women with coronary artery disease that did not demonstrate benefit for coronary or cerebrovascular events [14]. The pivotal Women’s Health Initiative (WHI), a primary prevention trial of estrogen plus progestin in healthy postmenopausal women, was stopped early due to overall risks, including breast cancer effects; it demonstrated an increased risk of stroke in the hormone replacement group [15]. These results precipitated a sharp and sustained decline in menopausal hormone therapy use [16].

Subsequent research highlighted chronological age, years since menopause, route, and dose as important modifiers of stroke risk from exogenous estrogens. In the WHI trial, the average age was well above 60 years [15]. In post hoc analysis, the risk of stroke was not significantly increased when women with prior cardiovascular disease and those older than 60 years were excluded [17]. The critical window hypothesis posits that prolonged estrogen deprivation alters estrogen receptor expression leading to a paradoxical pro-atherogenic response to estrogen in the late post-menopausal period [18]. In addition to timing, route of administration plays an important role. Exogenous oral estrogen undergoes the hepatic first pass effect with supraphysiologic hepatic exposure triggering prothrombotic and inflammatory changes. Transdermal estrogen may treat vasomotor symptoms without the same stroke risk as oral formulations by avoiding hepatic first pass metabolism [19]. Vaginal estrogen for treating local genitourinary symptoms has not been associated with increased stroke risk [20].

Despite increased stroke risk, hormonal therapy (HT) remains the most effective treatment for moderate to severe vasomotor symptoms. Progesterone should be added with estrogen in women with a uterus to reduce risk of endometrial hyperplasia. Decisions regarding HT must consider potential risks and benefits, and individual preferences should be honored. The ideal candidate for menopausal HT is < 60 years of age, within 10 years of menopause onset, at lower risk for cardiovascular disease, stroke, and breast cancer, and without other hormonal contraindications [21]. The 2024 Guideline for the Primary Prevention of Stroke states that oral estrogen-containing HT is associated with harm in women >/= 60 years of age, more than 10 years after non-surgical menopause, or at elevated vascular disease risk [21]. Nonhormonal treatment (e.g., cognitive behavioral therapy, serotonin reuptake inhibitors, neurokinin receptors (NKR) antagonists) should be prioritized for women at increased stroke risk [22]. NKR antagonists are a newer class of medications that target the thermoregulatory function of the hypothalamus [23].

Menopause timing and symptoms also impact cerebrovascular health. The Framingham Heart Study demonstrated that non-surgical menopause before 42 years of age doubled the risk of ischemic stroke, a finding that has been replicated [24–26]. The 2024 Guideline for the Primary Prevention of Stroke recommends screening for premature ovarian insufficiency (POI, before 40 years of age) and early menopause (before 45 years of age) to inform stroke risk. For individuals with POI or early menopause, evaluation and modification of vascular risk factors is recommended to reduce stroke risk [21]. Whether these patients benefit from HT to reduce long-term cardiovascular risk is unknown [21]. Vasomotor symptoms are associated with increased stroke risk but disentangling confounding effects is difficult as women with frequent, severe vasomotor symptoms also have more traditional cerebrovascular risk factors [27, 28].

## Migraine

Migraine disproportionately affects women, with a female to male prevalence ratio of three-to-one [29]. Migraine onset peaks during the reproductive years in women [30]. Reproductive hormones influence susceptibility of women to migraine, and migraines change in frequency and severity during reproductive events throughout a woman’s life. A significant portion of premenopausal women with migraines have menstrual migraines. Menstrual migraines occur in the pre/perimenstrual phase when estrogen levels fall precipitously in the transition from the luteal to follicular phase [31]. Migraine without aura is more closely linked to hormonal changes than migraine with aura. The estrogen withdrawal hypothesis is a proposed mechanism for menstrual migraines [32].

Many women experience migraine exacerbation during the perimenopausal period. In the fully adjusted model in the American Migraine Prevalence and Prevention study, high frequency headache was more common in perimenopausal but not postmenopausal women, compared to premenopausal women, supporting that the hormonal milieu and not aging, drives the headache burden [33]. As with menstrual migraines, the estrogen withdrawal hypothesis may explain perimenopausal exacerbation, which is a time of unpredictable and sometimes erratic estrogen levels, including rapid drops. Estrogen receptors are located in areas of the brain involved in pain perception and modulation [34]. Calcitonin gene-related peptide (CGRP) release is a key component of a migraine attack, and medications that suppress CGRP signaling are effectively treat migraines. Estrogen receptors are co-expressed with CGRP receptors, and rapid estrogen withdrawal leads to increased local CGRP release in pain-processing regions, promoting trigeminal sensitization and vasodilation [35, 36]. Oxytocin, regulated by estrogen, has anti-nociceptive properties and may also have an important role in CGRP release and migraine pathophysiology [37]. Progesterone likely also contributes to perimenopausal migraine but its role is a topic of ongoing research [37]. Premenopausal bilateral oophorectomy (surgical menopause) is associated with an increased risk of migraine; however, exogenous estrogen therapy likely plays a role in migraine occurrence in this population [38, 39].

The menopausal transition coincides with other life changes that can exacerbate migraine burden. Sleep disturbances, hot flashes, and other vasomotor symptoms may act as migraine triggers, contributing to perimenopausal exacerbation. Women with migraine may be more likely to experience severe vasomotor symptoms, which magnifies symptom burden and disability [40]. Age-related co-morbidities and medication overuse may also contribute to headache burden in postmenopausal women.

Many patients with migraine have a subsequent improvement following the last menstrual period (LMP) when hormone levels remain stably low without fluctuations. However, one population study found that nearly half of women with migraine continued to experience migraines following menopause and one in five had migraines persist after 60 years of age, which highlights the need to optimize migraine management for older women [41]. Migraine phenomenology in older adults may differ from younger individuals [42]. Migraine with aura, including aura without headache, is more likely following menopause. Migraine aura independently increases the risk of ischemic stroke and other vascular events [41, 43, 44].

Hormone therapy is a theoretical approach to prevent perimenopausal migraine; however, evidence remains limited and inconsistent. Some women with hormonally sensitive migraines report headache improvement, particularly with transdermal estrogen. Worsening of migraines with HT is reported most often with higher dose and oral formulations of estrogen [45–47]. HT may be used to treat moderate-to-severe vasomotor symptoms in women with migraine who are within 10 years of menopause and younger than 60 years of age without other contraindications, following individualized assessment of risks and benefits [48]. Migraine with aura is a risk factor for ischemic stroke so must be considered among the risks. In migraine patients, low-dose transdermal estrogen may be preferred as it provides a more consistent level of estrogen and has a lower risk of adverse thromboembolic and vascular events [44]. Standard migraine treatments remain applicable for peri- and post-menopausal women, although age-related co-morbidities impact safety and tolerability for many patients. Anti-CGRP therapies have emerged as first-line treatment for migraine. While early CGRP-targeted medication trials excluded older adult participants, more recent trials and real-world data suggests efficacy and safety in the post-menopause age range [49–52].

## Epilepsy

Epilepsy occurs at approximately equal rates in men and women. Sex steroid hormone levels throughout the life course, however, can affect seizure susceptibility in women with epilepsy (WWE). In premenopausal women with catamenial epilepsy, seizures cluster during the menstrual cycle due to fluctuations in ovarian steroid hormone levels. In general, estrogens have a proconvulsant effect and progesterone an inhibitory effect.

In one study, approximately two-thirds of WWE reported increased seizure frequency during perimenopause [53]. During the perimenopausal period, a shift from ovulatory to predominately anovulatory cycles occurs. During anovulatory cycles, the cyclic progesterone elevation that occurs in the luteal phase of ovulatory cycles does not occur. During perimenopause, loss of ovarian reserve leads to declining inhibin B levels and loss of negative feedback on FSH production. Estrogen levels are unpredictable and surge erratically in response to elevated FSH [54]. Elevated estrogen-to-progesterone ratios contribute to seizure tendency, particularly in women with a history of catamenial epilepsy, who may have increased vulnerability to fluctuating hormonal levels [55]. Vasomotor symptoms, particularly sleep disturbance, may worsen seizure control. For women with a history of catamenial epilepsy, seizure control tends to improve in the postmenopausal period as estrogen levels steadily and reliably decline [53]. Postmenopausal seizure course for WWE is widely variable [56]. Whether seizure outcomes after menopause differ between generalized and focal epilepsies is largely unknown, and syndrome-specific prognostication is limited [57].

WWE have an increased risk of POI [58]. The risk is greatest in women with frequent seizures; women with rare seizures seem to have a roughly equivalent age at menopause compared to women without seizures [59]. Seizure-induced hypothalamic-pituitary-gonadal axis dysfunction may lead to POI. Seizures, particularly those arising from temporal lobe structures, may disrupt gonadotropin-releasing hormone (GnRH) function in the hypothalamus and alter follicle-stimulating hormone (FSH) and luteinizing hormone (LH) release [60, 61]. This may result in dysregulation of ovarian follicle maturation and potentially early loss of follicles available for ovulation. Lifetime use of multiple anti-seizure medications may also contribute to early menopause [62].

Evidence regarding the impact of menopausal HT in WWE is limited [63]. In a small randomized controlled trial, HT (conjugated equine estrogen and medroxyprogesterone acetate) was associated with a dose-related increase in seizure frequency in postmenopausal WWE [64]. The progesterone formulation in HT may be an important consideration, and natural progesterone theoretically provides greater anticonvulsant properties [55]. Exogenous estrogen can decrease concentrations of some anti-seizure medications, particularly lamotrigine. Cytochrome P450 inducers can accelerate metabolism of exogenous oral HT and render it less effective for menopausal symptoms. Transdermal hormone therapy that bypasses hepatic metabolism may potentially be a preferred option, although data is lacking. Further studies are needed to fully understand the impact of menopausal HT on seizures. Clinicians should be vigilant around the time of starting HT in WWE.

To promote healthy aging among WWE, clinicians should recognize the need for close monitoring of bone health, sexual function, and vascular risk factors from compounded vulnerability from epilepsy, menopause, and anti-seizure medication exposure. Postmenopausal women with epilepsy are particularly vulnerable to osteoporosis and fractures [65]. Anti-seizure medication use and epilepsy itself are both associated with lower bone mineral density, and bone loss may begin before menopause [66, 67]. A wide range of anti-seizure medications, including both enzyme-inducing and non-enzyme-inducing, have been associated with lower bone health [67–71]. The US Preventive Services Task Force (USPSTF) and American College of Obstetrics and Gynecology (ACOG) recommend bone density screening in all postmenopausal patients >/=65 years and in younger patients with risk factors, with ACOG explicitly listing “antiepileptic drugs” as a risk factor for osteoporosis [72, 73]. Patients should be counseled to consume the recommended daily allowance of dietary calcium and vitamin D and receive monitoring and supplementation as needed [74].

## Multiple Sclerosis

Multiple sclerosis (MS), a CNS demyelinating condition, has a female-to-male incidence ratio of three-to-one [75]. Life expectancy of persons with MS has increased significantly since the introduction of disease-modifying therapies and with improved management of co-morbidities. With these advancements comes the need to better understand the influence of aging, including menopause, on disease course [76]. Over two-thirds of new MS diagnoses occur in premenopausal women [77]. Currently, about 30% of the population living with MS are peri- or postmenopausal women [77]. MS management during menopause is an important knowledge gap and has been identified as a significant priority [78–80]. For instance, clinical trials often restrict enrollment by age (< 50 or < 55 years) thus limiting our understanding of the efficacy of MS treatments in postmenopausal women [77]. In a recent global survey, MS patients rated menopause as a top priority area for research [79]. MS is influenced by other hormonal exposures, including puberty, pregnancy, and the postpartum period. Prepubertal MS is rare and, unlike MS across all age groups, prepubertal MS has a sex ratio close to one-to-one [81]. Due to immunological changes, pregnancy is often a time of relative quiescence for MS activity, followed by increased activity postpartum [82, 83]. Similarly, menopause may modulate the MS disease course [84].

MS is typically delineated into relapsing-remitting and progressive phenotypes; however, even in clinically relapsing-remitting cohorts, clinical relapses are superimposed onto an underlying neurodegenerative process. Following menopause, a relapse-progression paradox emerges, wherein relapse rates may decline while disability accelerates [80, 85, 86]. Progression independent of relapse activity (PIRA) describes chronic neurodegeneration that occurs separate from relapse-associated-worsening [87]. Aging-related immunosenescence contributes to reduced annualized relapse rates [88]. Meanwhile, inflammaging, chronic low-grade inflammation in older adults, accelerates the neurodegenerative process [89]. CNS repair and remyelination becomes less robust and iron accumulates with aging [89].

Menopause may represent an inflection point in accumulation of neuronal injury and functional decline in MS; however, studies report mixed results [85, 90–95]. For postmenopausal patients, disentangling the effects of reproductive aging (declining sex steroid hormones) from chronological aging (somatic immunosenescence) and disease duration is challenging. A lower annualized relapse rate (ARR) but worsening Expanded Disability Status Scale (EDSS) following menopause was found in some but not all studies [85, 94]. The EDSS is heavily weighted toward ambulation and other neurologic domains are poorly captured. Menopause may be an inflection point for biomarkers of neuronal injury, and this may be reflected on more granular scales, such as the multi-dimensional MS Functional Composite [90]. Biomarkers of ovarian functional decline, such as anti-Müllerian hormone, may help differentiate reproductive and somatic aging [96]. Changes in steroid hormone levels may independently interfere with neural repair mechanisms. High levels of endogenous estrogen are thought to be neuroprotective in MS, so its decline may account for the potential menopausal inflection point [97, 98]. Additionally, the transition to PIRA has important implications for therapeutics as many MS disease modifying therapies target relapse prevention.

Similarities between MS and perimenopausal symptoms may create a clinical diagnostic challenge. Sleep disturbances, fatigue, mental fogginess, depression, anxiety, and sexual and urinary dysfunction are common in both circumstances. Heat intolerance may trigger the Uhthoff phenomena, and vasomotor symptoms may trigger pseudo-relapse, significantly affecting quality of life [80]. Underlying MS does not clearly influence the average age of menopause onset or increase the risk of POI but specific disease-modifying therapies are known to affect ovarian function [99].

The evidence regarding HT’s impact on disease course and quality of life in postmenopausal women with MS is limited but growing. A phase Ib/IIa randomized controlled trial of HT for peri/postmenopausal women with MS and symptomatic hot flashes suggested good treatment tolerability [100]. A Danish nationwide cohort study suggested that HT did not affect disease activity nor disability progression in women with MS, when used for < 5 years [101]. Experiments in animal models of MS (autoimmune encephalomyelitis) suggest that exogenous hormones may prevent gray matter atrophy [102]. Recruitment for human clinical trials is hampered by perceived risks of HT [100]. Whether HT impacts MS progression in humans requires further research.

## Parkinson’s Disease

Parkinson’s Disease (PD), a neurodegenerative synucleinopathy, occurs with an approximately two-to-one male-to-female ratio [103]. Women tend to be diagnosed later and have less severe PD. Sex differences stem from multiple factors, including environmental exposures, hormonal milieu, genetic make-up, and immune function [104, 105]. Women mostly develop PD after menopause but continue to have a lower risk than men throughout the postmenopausal period. Steroidal hormone exposure is postulated to modify PD risk and disease course. Postmenopausal women with PD have lower estradiol and higher testosterone levels than controls; residual estradiol production is associated with milder motor symptoms and lower dopaminergic medication requirements [106].

Timing of menopause may impact risk and severity of PD. Some studies found that a later age at menopause may be associated with decreased risk of PD in women [107–109]. Conversely, earlier menopause may increase risk [110, 111]. However, findings are not uniform; one meta-analysis of observational studies did not find an association between age at menopause and PD risk; however, a subsequent meta-analysis did show an inverse relationship [112, 113]. Surgical menopause, especially bilateral oophorectomy prior to the age of 45 years, has been associated with increased risk of parkinsonism and PD [110, 113].

Clinical studies have investigated the role of HT on PD onset and symptomatology. Evidence regarding HT’s effect on parkinsonian symptoms is limited but suggests potential benefit for motor symptoms. A handful of studies have suggested that estrogen replacement may improve total and motor Unified Parkinson’s Disease Rating Scale (UPDRS) scores without significantly altering dyskinesias in postmenopausal women with PD but another pilot study found no impact [114–116]. A meta-analysis of 21 articles suggested that estrogen replacement may decrease risk of onset and progression of PD in postmenopausal women [117]. However, a retrospective study of a nationwide population-based cohort study in Korea suggested that hormone therapy was associated with an increased risk of PD in a regimen and duration-specific manner, and another nationwide Danish cohort found a nonsignificant increased risk with postmenopausal HT [118]. In general case-control studies of HT in PD tend to show an inverse (protective) association between HT and PD risk whereas cohort studies appear to show no or positive association. One potential explanation relates back to the critical window hypothesis. Early replacement of estrogen may be protective against dopaminergic degeneration, but the evidence is insufficient, and clinical practice guidelines do not currently recommend estrogen therapy for PD.

As with other neurological conditions, perimenopausal symptoms may overlap with the non-motor symptoms of PD, such as sleep disturbances, mood symptoms, cognitive changes, fatigue, and autonomic dysfunction. Physical activity has a beneficial role in PD and should be emphasized for postmenopausal women with PD.

## Alzheimer’s Dementia

Alzheimer’s Disease (AD), a neurodegenerative disease characterized by an abnormal accumulation of amyloid plaques and tau tangles, disproportionately affects women. The lifetime risk of AD is approximately double in women compared to men [119]. While longevity contributes to the higher prevalence in women, disparity persists after controlling for life expectancy [120]. The reasons are multifactorial and include impacts of sex steroidal hormones, interactions of sex with apolipoprotein E (APOE) alleles, amyloid, and tau, and differences in modifiable risk factors.

The APOE-e4 genotype, which can accelerate β-amyloid deposition, is the strongest known genetic risk factor for late-onset AD; the frequency of the APOE-e4 allele does not differ by sex, but its presence may confer a higher risk for developing AD earlier in women compared to men [121, 122]. Additionally, sex influences the interplay between β-amyloid and phosphorylated tau. In the presence of β-amyloid, women, particularly carriers of APOE-e4, accumulate tau pathology at an accelerated rate and may have a faster rate of cognitive decline [123, 124]. This also has potential implications for AD treatment. Amyloid-directed monoclonal antibodies for AD may potentially provide less benefit in women due to differential tau accumulation [125–127].

Endogenous estrogen has many potential neuroprotective effects including promoting neuronal growth and synaptic plasticity, regulating glucose metabolism, protecting against oxidative stress, inhibiting neuroinflammation, and preventing amyloid accumulation [128, 129]. The menopausal transition and accompanying estrogen decline impacts estrogenic regulation of brain glucose metabolism and can yield a hypometabolic state in AD-vulnerable brain regions [130]. Preclinical models suggest that the estradiol/progesterone imbalance also affects metabolic activity and energy availability [131]. The Bioenergetic Crisis Model proposes that menopausal estrogen decline triggers a cascade of metabolic adaptations that ultimately leads to amyloid and tau accumulation and AD pathology [132]. Perimenopause cognitive symptoms are common; however, objective cognitive decline is certainly not ubiquitous among individuals with perimenopausal cognitive symptoms. Other perimenopausal symptoms, such as insomnia and mood changes, impact perceived cognitive performance. Additionally, perimenopausal metabolic changes may be transient. Research suggests that some changes in brain regions observed during perimenopause stabilized or recovered in the postmenopausal group, suggesting adaptive compensatory processes [133].

Premature estrogen deficiency may trigger or exacerbate an underlying propensity for AD. Spontaneous premature ovarian insufficiency and early menopause are associated with accelerated cognitive decline and increased risk of dementia [134, 135]. Premenopausal oophorectomy (surgical menopause) is also associated with increased cognitive decline and risk of dementia [136–138]. Women with additional AD risk factors, such as APOE-e4 carriers, are particularly vulnerable to AD with early bilateral oophorectomy [136]. ACOG recommends systemic hormone therapy as an effective approach for symptoms of hypoestrogenism in POI and to reduce long-term health risks in patients without contraindications [139]. The 2025 American Society for Reproductive Medicine (ASRM) guideline further adds that health care professionals and women should know that POI is associated with an increased risk of cognitive impairment and dementia. They recommend HT in women with POI (without contraindications to HT) until the usual age of menopause to reduce the possible risk of cognitive impairment and dementia. Women with POI should also be counseled regarding lifestyle factors that can modify cognitive outcomes [140].

Additionally, investigators have explored the role of postmenopausal estrogen replacement in women with typical age of menopause. The Women’s Health Initiative Memory Study (WHIMS) enrolled women aged 65 years or older without probable dementia and randomized them to estrogen plus progestin versus placebo. The study found that the hormone replacement recipients had an increased risk of a subsequent diagnosis of probable dementia compared to the placebo group [141]. However, the trial’s applicability to younger women nearer to menopause is limited. As per the critical window hypothesis, the timing of estrogen may determine if it has neuroprotective, neuro-deleterious, or null effect. Subsequent trials of women within a few years of the LMP demonstrated no treatment-related cognitive benefit nor harm, which were reassuring for safety of HT [142, 143]. Recent meta-analyses suggest that estrogen therapy initiated during the critical window of the menopausal transition may be neuroprotective but further research is needed [144, 145]. The healthy cell bias posits that estrogen is neuroprotective for healthy neurons but accelerates neurodegeneration in diseased neurons by precipitating metabolic activity that exceeds the capacity of the unhealthy mitochondria [146]. Current evidence does not support prescribing HT solely for dementia prevention when menopause occurs at a typical age [147].

## Conclusions

Menopause has significant implications for the CNS conditions reviewed here: stroke, migraine, epilepsy, MS, PD, and AD; however, important unanswered questions remain. Estrogen is the most well characterized component of the menopausal hormonal changes impacting neurological disease. However, other hormones are known to play important roles, and metabolic, vascular, immunologic, and other changes are also crucial actors.

Data on the risks and benefits of HT for postmenopausal brain health are complicated and often contradictory. The effects depend on the specific neurological condition, patient age, time since menopause, route, dose, formulation, and co-morbidities. The strongest neurologic support for using HT is to prevent cognitive impairment and dementia following POI, until the usual age of menopause. Conversely, as it relates to stroke, oral HT is associated with harm in women >/= 60 years of age, more than 10 years after non-surgical menopause, or at elevated vascular disease risk. For all perimenopausal women, decisions regarding HT should take an individualized approach, weighing risks and benefits. Transdermal estrogen may impart a lower procoagulant effect through avoiding the hepatic first pass effect and may provide more consistent levels than oral formulations. The critical window hypothesis posits that HT may have neuroprotective effects early post menopause that dissipate or become harmful in later years.

Challenges for menopause-related neurologic research include limitations in translation animal models as few species undergo menopause as humans do. Recruitment for clinical trials involving HT is often slowed by high perceived risks among the public. Additionally, significant confounding by route, dose, formulation, and timing may occur. Menopause should be a research priority area for the field of neurology.

## Key References

- Haddadan, K.G., et al., *Recurrent ischemic stroke and vaginal estradiol in women with prior ischemic stroke: A nationwide nested case-control study.* Stroke, 2025. **56**(10): p. 2888–2894.
  - ○ Findings from this study suggest that vaginal estradiol does not increase the rate of recurrent stroke in women with a history of ischemic stroke.
- Bugge, N.S., et al., *Migraine through puberty and menopausal transition-data from the population-based Norwegian Women and Health study (NOWAC).* J Headache Pain, 2025. **26**(1): p. 145.
  - ○ Findings from this study suggest that migraine cessation occurs in approximately 80% of women before 60 years of age, and migraine with aura is more likely to persist after menopause.
- Bovenzi, R., et al., *A biological characterization of patients with postmenopausal Parkinson’s disease.* Journal of Neurology, 2024. **271**(6): p. 3610–3615.
  - ○ Findings from this study suggest that postmenopausal PD patients have lower estradiol levels compared to age-matched controls, and residual estradiol production is associated with milder motor symptoms and lower dopaminergic medication requirements.

## Data Availability

No datasets were generated or analysed during the current study.
